# Household

**Published:** 1890-02

**Authors:** 


### HOUSEHOLD.

Apples au Beurre.—Peel and core large apples; cut slices of stale bread about one-quarter of an inch in thickness, and then cut them again of a round shape, with the paste-cutter, about the size of the apples; spread some butter on each slice and place an apple on each; butter a baking pan, place the apples and bread in, fill the hole made in the middle of the apple with sugar; place on the top of the sugar a piece of butter about the size of a hazelnut, and then set them in a warm, but not quick oven; when about half done, fill up the holes again with sugar and a pinch of cinnamon, place butter on top as before, and finish the cooking. Glaze over with apple jelly, and put back in the oven for two minutes before serving. Serve hot.

Apple Bread.—A very light pleasant bread is made in France by a mixture of apples and flour, in the proportion of one of the former to two of the latter. The usual quantity of yeast is employed as in making common bread, and is beaten with flour and warm pulp of the apples after they have been boiled, and the dough is then considered as set; it is then put in a proper vessel and allowed to rise for eight or twelve hours, and then baked in long loaves. Very little water is requisite; none, generally, if the apples are very fresh.

Doughnuts.—Two eggs, two cupfuls each of sugar and new milk, one tablespoonful of thick cream, half a teaspoonful of salt, one teaspoonful of cinnamon or half of a nutmeg. Sift twice into a quart and a half of flour, three teaspoonfuls of cream of tartar, and one and a half teaspoonfuls of soda. Of the flour so prepared add enough to make a dough that can be handled. Let it rise one hour in a warm room, then roll out and fry in boiling lard. In most cases it is well to put the lard over the fire some time before it is needed, and put into it three or four pared raw potatoes cut in slices, removing them, of course, before frying the doughnuts. This does away with the objectionable taste of new lard.

Mountain House Roll.—This is a famous North River recipe, named from the Catskill Mountain House, where these rolls were first served. Set a thin sponge with wheat flour at about four o'clock as follows :—Stir into a quart of water flour enough to make a thick batter, adding half a cake of compressed yeast dissolved Let this sponge stand till nine o'clock and then knead up thoroughly; add a piece of butter the size of a large egg. Let the rolls stand till morning. then roll them out as thin as your hands, handle the dough as little as possible, cut it into narrow strips and lay in a pan to rise for three-quarters of an hour. Bake in a quick oven ten minutes. These rolls are delicious made of whole wheat flour.

Health Notes, 1.—In all cases of infectious disorder the attendants should avoid standing in a current of air which passes over the patient; they should avoid also inhaling the patient's breath, and moreover should not enter the room in the morning with an empty stomach, that is, fasting.

2. If the nail of the great toe is hard and apt to grow round and into the corners of the toe, take a piece of broken glass, and scrape the top very thin; do this every time you cut your nails, and by constant use it makes the corners fly up and grow flat, so that it is impossible that they will give any pain.

